# Acceptability, usability and feasibility of experienced sampling method in chronic secondary pain syndromes

**DOI:** 10.3389/fneur.2023.1219236

**Published:** 2023-07-12

**Authors:** Aysun Damci, Janneke G. J. Hoeijmakers, Marlies den Hollander, Albère Köke, Marion de Mooij, Catharina G. Faber, Jeanine A. M. C. F. Verbunt

**Affiliations:** ^1^MHeNS, School for Mental Health and Neuroscience, Maastricht University, Maastricht, Netherlands; ^2^Department of Neurology, Maastricht University Medical Center+, Maastricht, Netherlands; ^3^Care and Public Health Research Institute, Maastricht University, Maastricht, Netherlands; ^4^Department of Rehabilitation Medicine, Maastricht University Medical Center+, Maastricht, Netherlands; ^5^Adelante Zorggroep, Center of Rehabilitation Medicine, Hoensbroek, Netherlands,; ^6^Dutch Network Pain Rehabilitation, Hoensbroek, Netherlands

**Keywords:** chronic pain, experience sampling method, small fiber neuropathy, feasibility, clinical trial

## Abstract

**Background:**

In chronic pain syndromes, symptoms can fluctuate and change over time. Standard questionnaires cannot register these fluctuations. Nonetheless, the experience sampling method (ESM) is developed to collect momentary measurements of everyday complaints, tracing fluctuations in symptoms and disabling factors over time. Although valuable information can be collected in this way, assessment may also be a burden. This study aimed to investigate the acceptability, usability, and feasibility of ESM in chronic secondary pain syndromes, in a single-center study in the Netherlands.

**Methods:**

A prospective observational study with repeated measurements was conducted in patients with chronic secondary neuropathic and musculoskeletal pain syndromes, including small fiber neuropathy, spinal cord injury, and rheumatoid disorder.

**Results:**

Thirty-four participants were included and filled in the ESM, of whom 19 were diagnosed with small fiber neuropathy, 11 with spinal cord injury, and 4 with a rheumatoid disorder. The mean age was 54.7 ± 13.9 years (range: 23–77) of whom 52.9% were female. In total, 19 participants filled in the general and user-friendliness evaluation about the acceptability and usability of the ESM. The general evaluation showed no influence of ESM on participants’ social contacts (mean 1.47, SD 1.12), activities (mean 1.74, SD 1.44), and mood (mean 1.89, SD 1.59). The answers options of ESM were a good representation of the experiences of participants (mean 4.58, SD 1.77). Regarding feasibility, the overall response rate for answering the beep signals of ESM was 44.5% in total. The missing rate per person varied from 13% to 97% with a median of 54.1%.

**Conclusion:**

The general evaluation and the user-friendliness revealed sufficient outcomes in favor of the ESM application. ESM seems a promising measurement tool to use in secondary chronic pain syndromes.

## 1. Introduction

Chronic pain can be a symptom of a primary pain syndrome, in which pain cannot be accounted for by another pain condition or underlying disease, or a secondary pain syndrome, in which pain is linked to other diseases as the underlying cause (1). Almost 18%–34% of adults in the United States and Europe are affected by chronic pain (2, 3). Physical functioning and quality of life (QOL) are often negatively affected in chronic pain syndromes (4, 5). As became clear in the last decades, pain-related disability has a biopsychosocial character. As we know from research in primary pain syndromes, (dynamic) psychosocial factors interact and interfere with daily functioning in chronic pain (6–8). Their joint impact potentially accounts for changes in pain intensity and pain-related disability (9). In secondary chronic pain syndromes, biomedical factors can even lead to an additional disabling impact. Fluctuations in somatic symptoms related to the underlying medical disorder also may negatively interact with psychosocial factors leading to a pain as well as daily functioning with a joined impact on pain intensity and disability (9–11). The treatment of the underlying disease in a secondary chronic pain syndrome will not automatically result in the improvement of pain intensity or QOL with disease management. However, the impact of pain is not always investigated with a biopsychosocial approach (12, 13). It is necessary to be aware of the pain management and the underlying biopsychosocial factors in order to unravel and understand fluctuations of all biopsychosocial factors over time and their impact on QOL, pain and pain-related disability to develop a personalized treatment approach (9, 11, 14–19).

Pain intensity, psychosocial factors (including catastrophizing, fear, anxiety, and depression) and biomedical factors (such as f.e., spasticity in spinal cord injury related pain or disease activity in rheumatoid arthritis) as well as daily activities are not stable but may fluctuate and change throughout the day (11, 20). Measuring changes in these factors within a day, (probably) influencing pain intensity and pain-related disability, is possible with a diary. This contrasts standard questionnaires, which cannot be used for the momentary assessment of biopsychosocial factors (21). Moreover, these questionnaires are not able to capture daily changes as they need the support of the memory to recall symptoms and pain over a certain period (22). As a result, retrospective assessments are prone to result in an over- or underestimation of complaints (15, 17, 23–27). In contrast, real-time changes can be assessed with a daily diary, which avoids recall and memory bias (27).

The experienced sampling method (ESM) is a smartphone-based diary (application), that gathers real-time repeated measurements at randomly determined moments in the natural environment of everyday complaints (9, 16, 22, 28–31). A more profound understanding of disease-related fluctuations, including mood, somatic symptoms, thoughts, and physical activity, can be monitored (11, 15–17). Several beep signals per day are sent as a reminder to fill in a set of questions and statements for a consecutive period, which could result in a burden. In order to measure the fluctuations of the biopsychosocial factors with ESM, a feasibility study has to confirm the acceptability, usability and feasibility of ESM. Various feasibility studies have proven ESM to be valid and reliable in the daily-life assessment of primary chronic pain (9, 15, 17, 32). However, several predictors have been clarified, negatively influencing the response rate, including male gender, younger age, substance abuse, and negative affect (22, 33–36). There are also a couple of requirements for the successful use of ESM to prevent missing data, e.g., being able to use a smartphone and thinking of a low battery to charge in time (37). However, the exact addition of ESM data to the clinical practice remains unclear.

The aim of this study is to analyze the acceptability, usability, and feasibility of ESM in chronic secondary neuropathic pain syndromes and chronic secondary musculoskeletal pain syndromes, in a single-center study in the Netherlands.

## 2. Methods

### 2.1. Design

In Adelante location Maastricht University Medical Center+ (the Netherlands), an observational study with repeated measurements was conducted in participants with chronic secondary neuropathic pain syndromes and chronic secondary musculoskeletal pain syndromes, including small fiber neuropathy, spinal cord injury, and rheumatoid disorder. Adelante is specialized in the rehabilitation of patients with chronic pain syndromes, therefore, patients, living everywhere in the Netherlands, will come to the outpatient clinic to be treated. Participants were recruited in the outpatient clinic between September 2021 and September 2022. The following participants were included: age older than 18 years, who experience pain-related disability in daily life activities. Approval of the study was obtained by the medical ethical committee of Zuyderland Medical Center (METCZ20210022).

### 2.2. Device

**Figure 1:**
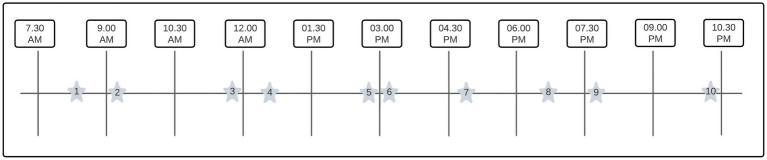
Example of a daily (random) beep scheme. A visualization of a random and time-contingent ESM schedule. The stars are indicating the random beep signals within a time window of 90 minutes.

To conduct an ESM study design, PsyMate^©^ (smart-eHealth GmbH, Luxembourg) has been used. PsyMate^©^ is a smartphone-compatible diary (Android and Apple) developed by the department of Psychiatry of the Maastricht University Medical Center+. A time- and signal-contingent schedule was set with 10 beep signals a day for 7 consecutive days between 7:30 AM and 10:30 PM (17, 29) because in other ESM studies, more than 7 beep signals resulted in a response rate of 86% (31). The beep signal schedule was randomly set and had an interval of at least 15 min and a maximum of 90 min between each beep signal, see Figure 1. Participants had to fill in 18 questions including biomedical (specifically related to their disease), mood, psychological, and social topics. An overview of the ESM questions is summarized in Table 1. After the beep signal, the participant had 15 min to fill in the questions and statements, otherwise, the questions disappeared and became missing values. Participants were asked to complete all questions and statements.

**Table 1:** Overview of experience sampling method (ESM) questions with scoring.

ESM question	
I am in pain	0 = no pain, 10 = worst pain
I am tired	0 = not tired, 10 = very tired
I am cheerful	1 = not, 7 = very
I am relaxed	1 = not, 7 = very
I am sad	1 = not, 7 = very
I am anxious	1 = not, 7 = very
I was physical active since the last beep signal	1 = not, 7 = very
I want the pain to stop	1 = not, 7 = very
I am not thinking clear	1 = not, 7 = very
I try to move less	1 = not, 7 = very
The pain determines what I am doing	1 = not, 7 = very
What am I doing?	Work, housekeeping-related activities, food-related activities, selfcare, care for others, resting, social contact, nothing, use of social media, sports, relaxing, something else
Where am I?	Home, visitation, at work, public place, on my way to, somewhere else
Who is joining me?	No one, partner, family, friends, coworkers, acquaintances, strangers

On the last day of enrollment, participants were asked to fill in an acceptability and usability questionnaire for a general and user-friendliness evaluation, consisting of 18 questions with nine questions about the general evaluation, and nine questions about the user-friendliness. Acceptability is defined as appropriate use of the ESM tool from the participants’ perspective, usability as the ability to use the measurement tool ESM, and the feasibility is based on the implementation of the measurement tool in practice.

### 2.3. Procedure

**Figure 2:**
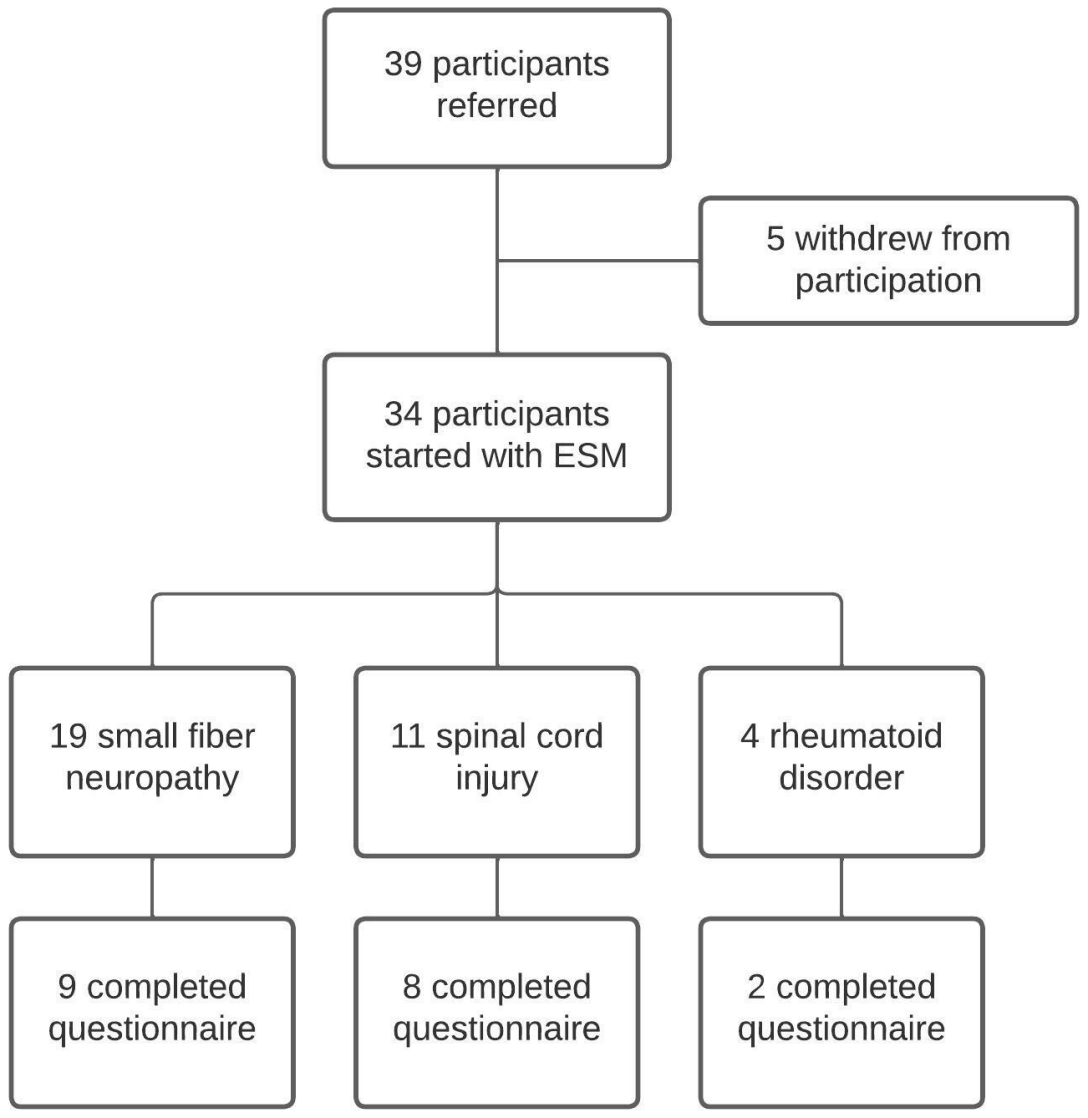
Inclusion flowchart of the study. In total 39 participants agreed to participate, of whom 3 withdrew due to lack of interest and 2 were not able to use the application due to smartphone-related technical issues. In total, 34 participants were included.

The rehabilitation physician checked the inclusion and exclusion criteria during a consultation in the outpatient clinic. After informed consent approval, eligible participants were contacted by the research team to provide additional study information. Sociodemographic information was collected with a questionnaire, including college degree, work state and substance abuse, such as tobacco, alcohol and drugs. Participants received information and instructions about the ESM application *via* phone contact. When experiencing any problems, they could call or e-mail the research assistant. Figure 2 shows the inclusion flowchart of the study.

### 2.4. Statistical analysis

The baseline variables were calculated as frequencies and proportions of categorical variables and means and standard deviations of continuous variables.

### 2.5. Acceptability and usability analysis

The acceptability and usability were based on the outcome of the general and user-friendliness evaluation (questionnaire). The questions and statements are presented in Table 2. The questions and statements were scored on a Likert scale of 7 (1 = not, 7 = very much), and, the overall satisfaction score was scored on a 11-point numeric rating scale (0 = not, 10 = very much). The outcome was calculated as frequencies and proportions.

**Table 2:** General and user-friendliness evaluation of PsyMate application (*N* = 19).

General and user-friendliness evaluation	Scores (1 = not at all, 7 = very much)
*General evaluation of PsyMate application, mean (SD; range)*	
Was this a normal week?	4.95 (1.87; 1–7)
Where there any special events this week?	3.32 (2.11; 1–7)
Were the questions a good representation of your experiences?	4.58 (1.77; 1–7)
Did the PsyMate influence your social contacts?	1.47 (1.12; 1–5)
Did the PsyMate influence your activities?	1.74 (1.44; 1–6)
Did the PsyMate influence your mood?	1.89 (1.59; 1–6)
Did the PsyMate hinder activities?	1.68 (1.60; 1–7)
Did you made mistakes answering the questions?	2.42 (0.90; 1–5)
Overall satisfaction score	5.06 (1.47; 2–7)
*Evaluation of user-friendliness of Psymate application, mean (SD; range), % (n)*	
Where the questions unclear or difficult?	2.68 (1.86; 1–6)
Where you able to read the text ton the screen well?	6.16 (1.46; 1–7)
Did you have any problems using the PsyMate application?	1.68 (1.60; 1–7)
Did you experience the use of the PsyMate burdensome with regard to the duration filling in of the questions?	2.42 (2.09; 1–7)
Did you experience the use of the PsyMate burdensome with regard to the sound?	2.16 (1.64; 1–6)
Did you experience the use of the PsyMate burdensome with regard to the number of the beep signals?	3.11 (2.08; 1–7)
Did you experience the use of the PsyMate burdensome with regard to carrying the smartphone the whole day?	3.11 (2.23; 1–7)
Did you experience technical issues?	
Yes	36.8% (7)
No	63.2%
Did these technical issues resolve?	
Yes	4.3% (1)
No, it took more than 1 day	4.3% (1)
Unsolved	73.9% (5)

### 2.6. Feasibility analysis

For feasibility, the overall response rate (compliance) was calculated on a daily and a weekly basis at the end of the survey as frequencies and proportions of each participant. The presentation of completion rates, ranges of completion rates, and distributions of completion rates were mentioned (38). An overall response rate of 33% was mentioned to be minimally required to analyze ESM data (39). Missing values were analyzed with a missing value analysis. The reason for missing data was analyzed: missing (completely) at random, and not missing at random (36, 40). The level of 30% was taken as a significant degree of missingness and resulted in a dichotomous variable. Thereafter, we studied whether missingness could be predicted by baseline characteristics by using a multilevel binary logistic regression (41). The following predictors were integrated into the regression analysis: age, gender (male-female), an education level (lower or higher than a college degree), work state (working or not), the outcome of hospital anxiety and depression scale (0–7, normal score, 8–10: borderline, and ≥11: abnormal), and substance abuse [yes (=1) or no (=0)] [tobacco, alcohol use, drugs (yes or no)] (22, 33–35). The results were analyzed as a total, and not according to the diagnosis.

## 3. Results

### 3.1. General baseline characteristics

Thirty-nine participants agreed to start with the PsyMate application, of whom three participants eventually withdrew before inclusion due to lack of interest, and two participants were not able to start with the application due to smartphone-related technical issues after the inclusion, and were excluded from further analysis, see Figure 2. In total, 34 participants were included to fill in the PsyMate application, of which 19 participants suffered from small fiber neuropathy, 11 had a spinal cord injury, and four were diagnosed with a rheumatoid disorder. The mean age of all participants (*n* = 34) was 54.7 ± 13.9 years (range: 23–77), of whom 52.9% were female. Twenty participants did not have paid work, of whom five participants were retired. Twenty-nine participants completed college and had at least a college degree. Regarding intoxication: 64.7% used alcohol (38.2% 1–3 units/week, 14.7% 4–7 units/week, 5.9% 8–14 units/week, and 5.9% >21 units/week), 8.8% used drugs [CBD with THC (*n* = 2), and XTC (*n* = 1)], and 13.9% used tobacco.

### 3.2. Acceptability and usability of ESM

**Figure 3:**
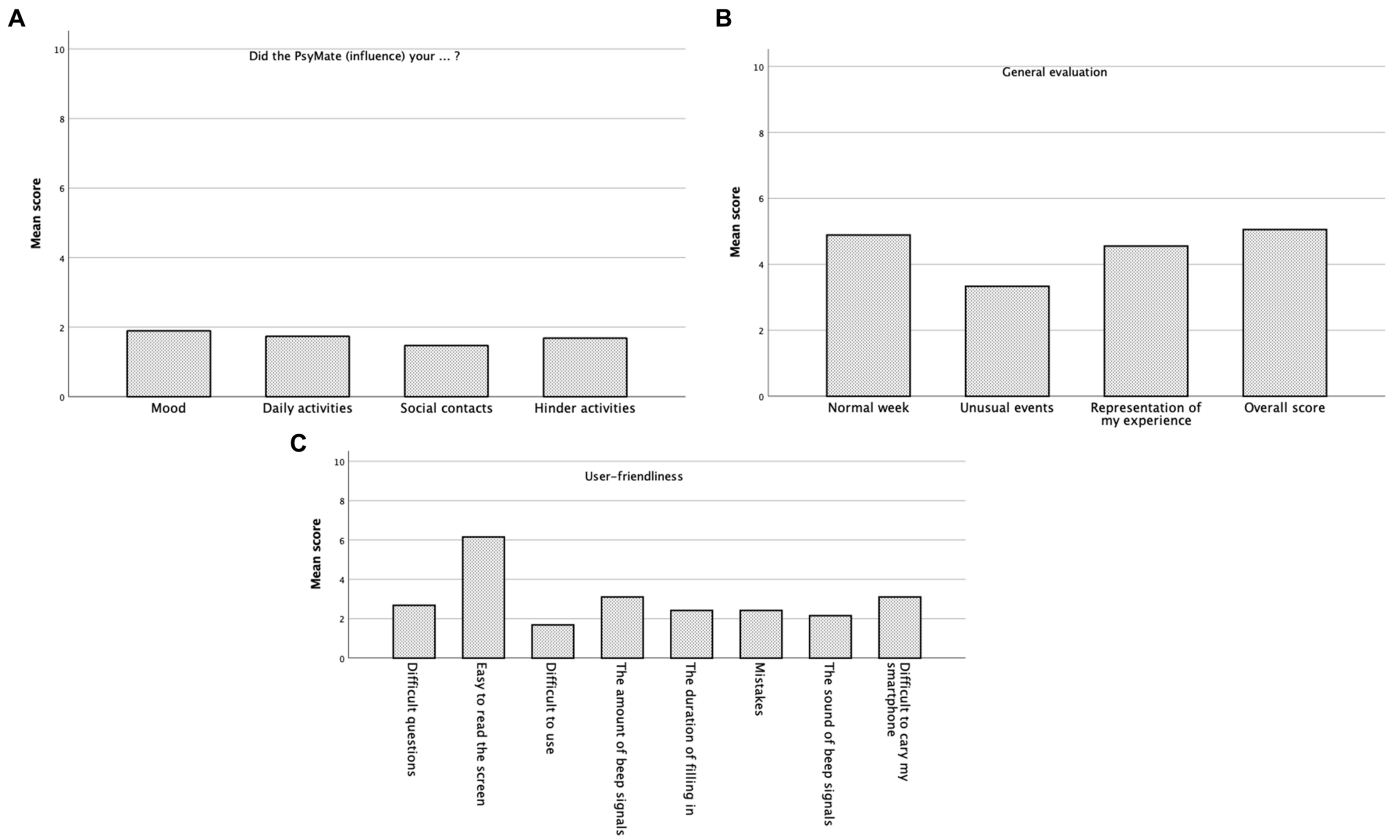
General and user-friendliness evaluation of PsyMate application. **(A-C)** The mean scores of the general and user-friendliness evaluation have been illustrated in a graphical design. All questions could be answered on a 7-Likert scale, only one question (overall score) could be answered on a 11-Likert-scale.

In total, 19 participants (of 34 participants) completed the general and user-friendliness evaluation at the end of their study participation. Therefore, the outcome of these 19 participants was analyzed. The findings were summarized, see Table 2 and Figures 3A–C. Most of the participants reported the study-period week to be a normal week (mean 4.95, SD 1.87). Participants indicated that the answers they could choose included a good representation of their experiences (mean 4.58, SD 1.77). PsyMate did not influence their social contacts (mean 1.47, SD 1.12), their activities (mean 1.74, SD 1.44), and their mood (mean 1.89, SD 1.59).

### 3.3. Feasibility characteristics

Of the maximum total of 2,380 beep signals that could be gathered (70 per participant, with a total of 34 participants), 1,058 responses were collected, indicating a response rate of 44.5% (compliance). The missing rate per person varied from 13% to 97% with a median of 54.1%. The number of missing beep signals varied between 9–69, with a median of 39.8 missing beep numbers per participant. The baseline characteristics of these participants were included in the analysis. The average response time of all participants (who filled in ESM) was 87.48 ± 56.49 s. No differences were found in the response rates between the different secondary chronic pain disorders.

### 3.4. Missing value analysis

**Figure 4:**
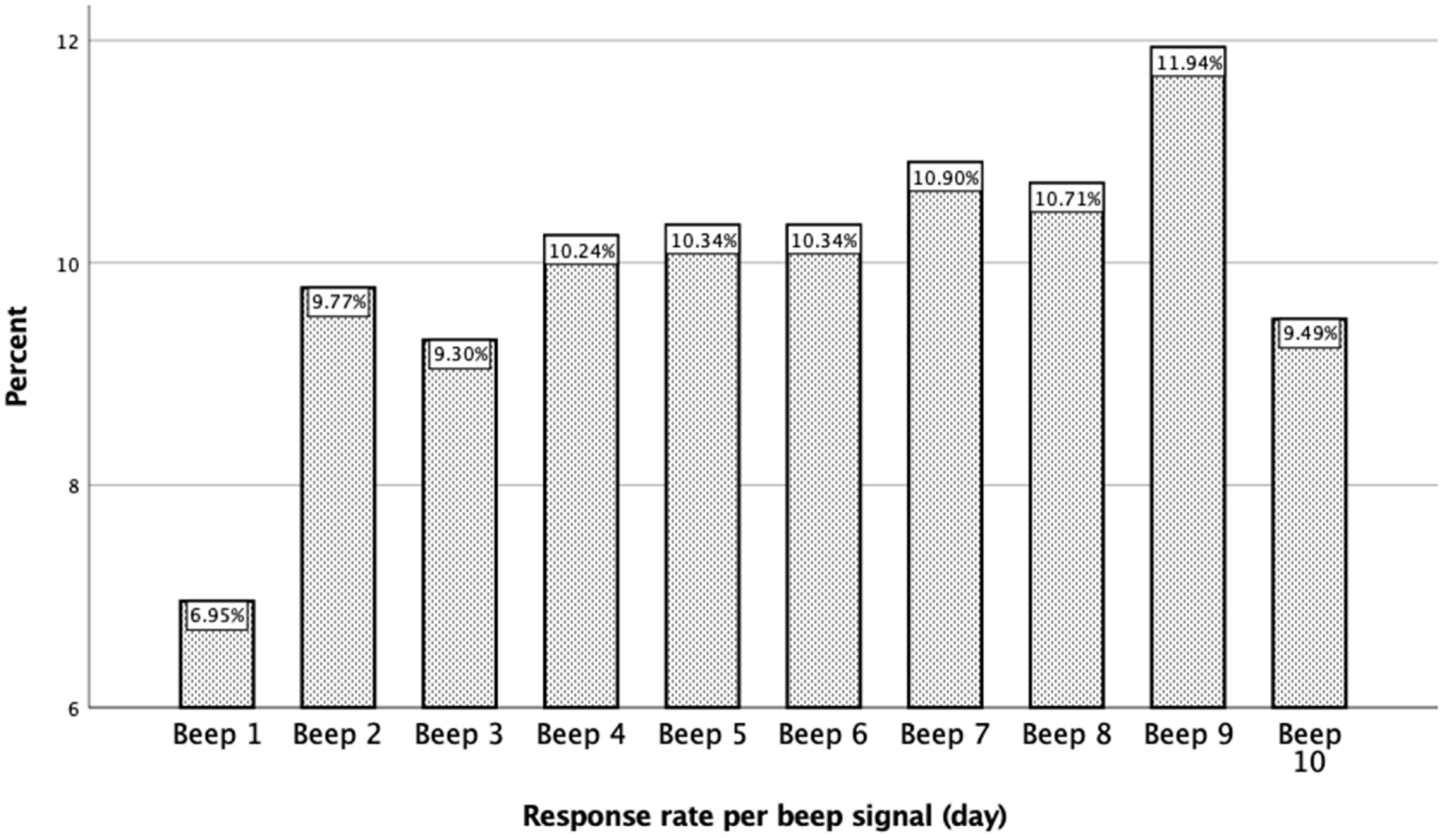
Overview of the percentage of completed beeps in a day.

The earliest and the latest daily beep signals were completed less frequently, 6.9% and 9.4% respectively, see Figure 4. We performed a binary logistic regression analysis in order to study whether a low response rate could be predicted; however, the analysis did not reveal any association [*p*-value >0.998, 95% CI (0.00–0.00)]. It appeared that the missing values were completely at random for each participant.

## 4. Discussion

In this study, we investigated the acceptability, usability, and feasibility, of ESM in 34 participants with chronic secondary neuropathic musculoskeletal pain syndromes. The overall response rate was 44.5%, with a range between 13% to 97%. Measuring acceptability and usability of revealed favorable outcomes for ESM.

In earlier ESM studies in participants with a psychiatric diagnosis or a primary chronic pain syndrome, the response rate (compliance) was reported to be between 71.1% and 86% (22, 32, 35). We found a response rate of 44.5%, which is above our pre-set minimum of 33% (39), but remarkably lower than the abovementioned samples. Several factors can be related to this difference: We will divide this into participant- or application-related issues. Participant-related issues will first be discussed. Both, a younger or older age, could result in (more) missing values. Younger participants are mostly carefree with less responsibility, and an increasing missing data over participation time (33–35). On the contrary, an older age could be accompanied by (more) technological difficulties, when compared with a younger age (42). In our data, the age range was 23–77 (mean 54.7 ± 13.9 years). Nonetheless, within our group of participants, we did not find any age-related association. Another explanation could be gender. It has been reported that missing rates are much higher in male participants (34). Forty-seven percent of our participants are males, but no association between the number of missing rates and male gender could be identified. Neither for the association between the missing rate and substance abuse (tobacco, alcohol, and drugs), negative affect, and educational level [*p*-value >0.998, 95% CI (0.00–0.00)] (22, 35, 43–45). Moreover, no differences were found between the three different secondary chronic pain disorders. We can cautiously indicate that participant-related factors probably did not influence the amount of missing data and completion rates.

As previously mentioned, application-related factors could also have contributed to missing data. Although, the number of 10 beep signals per day was reported to be feasible enough (29), however, why do we observe a low completion rate when compared to earlier ESM research? Only 44.5% of all questions and statements were filled in (1058/2520). This means that per participant, an average of 31 of the totals of 70 beep signals (10 beep signals per day for 7 days) were completed. Could ESM create an intrusive threshold? A high number of beep signals could create an intrusive threshold, resulting in a burden. The results of the general and user-friendliness evaluation reported however less interference when compared to scores when compared to other literature (32). Participants reported not experiencing any difficulties with the management of the application or reading the questions and statements on the smartphone. It thus seemed that filling in the ESM application was acceptable and usable. Another possible explanation could be technological difficulties. Only 7 participants experienced technical issues, mostly related to the operating system of their smartphones. Some operating systems hinder the signals of certain applications to maintain a longer-lasting battery level. For 5 participants, the issues could not be solved during and after the participation period. Solving technical problems was problematic due to the use of telephone contact and the distance between the participants and the research team. Therefore, if a participant experienced a technical problem, there is a great chance that the problems will remain unsolved. Overall, these results are indicating a favorable general and user-friendliness evaluation, that ESM might be feasible for patients with a secondary chronic pain syndrome.

Missing values might be missing (completely) at random and missing not at random (38, 40). To study the reasons for missingness, a binary logistic regression analysis was performed, but no pattern could be detected in the missing data. As a result of this analysis, we may conclude that our missing values are missing at random (40). However, we also observed that the earliest and the latest beep signals were the least filled in, 6.95% and 9.49% respectively, see Figure 4. The beep signals were planned according to a time-contingent, daily random scheme. A time window varying from 15 to 90 min was planned between each beep signal. Nonetheless, the first beep was between 7.30 AM and 9.00 AM, and the last beep (number 10) was between 9.00 PM and 10.30 PM, see also Figure 1. This finding could indicate that there are differences in biological wake-sleep rhythm among chronic pain participants, independently of work state (46). This may have implications for future studies in which ESM beep schemes will be used in chronic pain participants. Our results imply that this wake–sleep rhythm needs to be taken into account, e.g., the first beep a little later on the day, 8.30 AM–9.00 AM instead of 7.30 AM–9.00 AM.

There are some strengths and limitations accompanying this study. First, we will discuss the strengths. This is the first study investigating the acceptability, usability, and feasibility of ESM in secondary chronic pain syndromes. Moreover, our results are indicating that ESM could safely be used in secondary chronic pain syndromes. Regarding ESM itself, ESM seems to be less time-consuming. The response time on each beep signal was on average 87 s. In addition, participants reported not experiencing any difficulties related to ESM, indicating that ESM is easily manageable. Moreover, participants mentioned that ESM did not affect their mood, daily activities, and social contacts. It seems that participants are not adapting their daily and social activities to the use of ESM. A couple of limitations will also be discussed. Although, more than 7/10 beep signals were reported to be feasible (29), however, a low response rate is present in our data. Questions in an electronic diary should be brief. In our opinion, reducing the amount of beep signals could be more feasible, probably resulting in a higher response rate. However, crucial information about what happens between beep signals will be lost. As next, only 19 of the included 34 participants filled in the acceptability and usability questionnaire. This could indicate that a certain group of participants were motivated and probably experienced less problems with ESM, creating a selection bias among participants itself. Sending all participants, a paper version of the acceptability and usability questionnaire, could be a future solution to avoid a number of missing questionnaires and data. As last, another solution could be to solve technical issues faster, which is discussed earlier, such as solving technical issues physically rather than at a distance, or to offering a chatbot.

In conclusion, ESM is a promising measurement tool to help patients and healthcare professionals to gain more insight into daily fluctuations of pain and possible associations with pain-related thoughts, emotions, activities, and environmental factors for primary and secondary chronic pain syndromes, by offering visualizations of the complex disease-related dynamics for patients and healthcare professionals. Measuring the acceptability and usability revealed favorable and encouraging outcomes, with generally satisfied participants.

## Data availability statement

The raw data supporting the conclusions of this article will be made available by the authors, without undue reservation.

## Ethics statement

The studies involving human participants were reviewed and approved by Medical Ethical Committee of Zuyderland Medical Center (METCZ20210022). The patients/participants provided their written informed consent to participate in this study.

## Author contributions

AK and JV contributed to conception and design of the study. AD, JH, MH, AK, CF, and JV wrote the first draft and sections of the manuscript. AD organized the database and performed the statistical analysis. All authors contributed to the article and approved the submitted version.

## Funding

The present study was funded by the Prinses Beatrix Spierfonds [grant number W.OK17-09].

## Conflict of interest

The authors declare that the research was conducted in the absence of any commercial or financial relationships that could be construed as a potential conflict of interest.

## Publisher’s note

All claims expressed in this article are solely those of the authors and do not necessarily represent those of their affiliated organizations, or those of the publisher, the editors and the reviewers. Any product that may be evaluated in this article, or claim that may be made by its manufacturer, is not guaranteed or endorsed by the publisher.
